# Simple and Efficient Synthesis of Racemic 2-(*tert*-Butoxycarbon-ylamino)-2-methyl-3-(1*H*-1,2,4-triazol-1-yl)propanoic Acid, a New Derivative of β-(1,2,4-Triazol-1-yl)alanine

**DOI:** 10.3390/molecules16043380

**Published:** 2011-04-19

**Authors:** Younas Aouine, Hassane Faraj, Anouar Alami, Abdelilah El-Hallaoui, Abdelrhani Elachqar, Abdelali Kerbal

**Affiliations:** 1 Laboratoire de Chimie Organique Fès, Faculté des Sciences Dhar El Mahraz, Université Sidi Mohamed Ben Abdellah, Morocco; 2 Centre Universitaire Régional d’Interface (CURI), Université Sidi Mohamed Ben Abdellah, Fès, Morocco

**Keywords:** oxazoline, 1*H*-1,2,4-triazole, high regioselective alkylation, β‑aminoalcohol, β‑(1,2,4-triazol-1-yl)alanine

## Abstract

A simple synthetic approach to racemic *N*-*tert*-butyloxycarbonyl-2-methyl-3-(1*H*-1,2,4-triazol-1-yl)alanine (**5**) in four steps and 68% overall yield starting from oxazoline derivative **1** is reported. This synthesis involves the alkylation of 1*H*-1,2,4-triazole with an *O*-tosyloxazoline derivative, followed by an oxazoline ring-opening reaction and oxidation of the *N*-protected β‑aminoalcohol by potassium permanganate.

## 1. Introduction

Triazoles constitute an important class of biologically active heterocyclic compounds that have received a great deal of attention since their discovery. Diverse compounds derived from 1,2,4-triazoles have a wide spectrum activities, including antimicrobial [1,2] and antibacterial properties [3,4], human antifungal agents [5], anticancer agents [6], antiviral [7], antitumor activity [8], inhibitors of cytochrome P450 14α-demethylase (CYP51) [9] and in agricultural science as potent fungicides, herbicides and insecticides [10,11]. Amino acids containing the 1,2,4-triazole moiety and their derivatives represent a well-known group of organic compounds also presenting biological activity. Thus β-(1,2,4-triazol-1-yl)-L-alanine is known as an important metabolite in plants of the fungicide myclobutanil [12,13,14] and β-(3-amino-1,2,4-triazol-1-yl)-L-alanine is a metabolite of the weedkiller 3-amino-1,2,4-triazole [15]. Considering the interest in these heterocyclic amino acids, several structurally related nonproteinogenic amino acids and their derivatives have been the subject of various investigations. For example, preparation of methyl 2-(bis(*tert*-butoxycarbonyl)amino)-3-(1*H*-1,2,4-triazol-1-yl)-propanoate, a derivative of β-(l,2,4-triazol-l-yl)-alanine by a Michael addition of 1*H*-1,2,4-triazole to *N,N-*bis(*tert*-butyloxycarbonyl)dehydroalanine methyl ester has been described [16]. The same authors also described the synthesis of methyl 2-(*N*-(*tert*-butoxycarbonyl)benzamido)-3-(1*H*-1,2,4-triazol-1-yl)butanoate [17] according to the same reaction process mentioned previously.

Continuing our investigations in the use of oxazoline derivative in heterocyclic synthesis [18,19,20] we present herein a convenient and easy procedure for the preparation of racemic *N*-*tert*-butyloxy-carbonyl-2-methyl-3-(1*H*-1,2,4-triazol-1-yl)alanine, a new derivative of β-(1,2,4-triazol-1-yl)alanine.

## 2. Results and Discussion

**Scheme 1:**
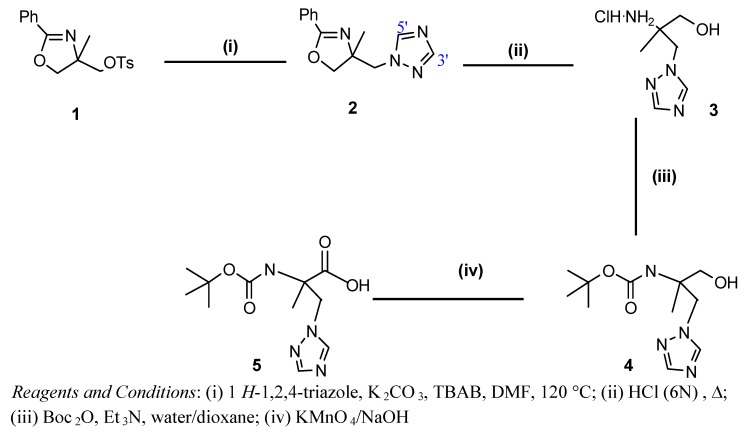
Strategy of synthesis of compound **5**.

Our strategy for the synthesis of *N*-*tert*-butyloxycarbonyl-2-methyl-3-(1*H*-1,2,4-triazol-1-yl)alanine **5 **is based on the substitution of the *O*-tosyl group present in the oxazoline ring with 1*H*-1,2,4-triazole (Scheme 1). It is reported that, the alkylation of 1,2,4-triazole with alkyl halides and a variety of bases afforded the corresponding 1- and 4-alkylated isomers, with prevalence of the N_1_-isomer [21,22,23]. Reaction of 1*H*-1,2,4-triazole with **1** and K_2_CO_3_, was carried out in the presence of a catalytic amount of tetrabutylammonium bromide in *N*,*N*’-dimethylformamide at 120 °C for 12 hours.

Application of our method to 1,2,4-triazole afforded only the 1-substituted product, and after column chromatography on silica gel we isolated only one isomer. Product **2** was obtained in 95% yield from **1** and was characterized by MS, ^1^H-NMR and ^13^C-NMR spectroscopy. The structure of N_1_-isomer **2** was assigned by comparison with the literature data [16,21,22,23] concerning the chemical shifts of triazole protons and the chemical shifts of the carbons of the triazole ring in positions 3' and 5' (see Scheme 1). Indeed, the ^1^H-NMR spectrum of **2** show two signals at 7.88 and 8.17 ppm for the two triazole protons (H^5'triazole^, H^3'triazole^) which are not equivalent. In the same way, the ^13^C-NMR spectrum of **2** also shows two signals at 151.42 and 144.32 ppm relating to the carbons of the triazole ring in positions 3' and 5'. 

The preceding reaction stage is followed by an oxazoline ring-opening reaction carried out in acidic medium. The aminoalcohol derivative **3** was obtained in 97% yield. The addition of Boc_2_O to the product **3** in a mixture of water/dioxane in the presence of triethylamine leads to *N*-protected-β-aminoalcohol **4** (yield 80%).

The action of dilute KMnO_4_ on compound **4** in basic medium (NaOH) led after four hours at room temperature to *N*-*tert*-butyloxycarbonyl-2-methyl-3-(1H-1,2,4-triazol-1-yl)alanine (**5**) in a yield of 92%. The structures of products **4 **and **5 **were established on the basis of NMR spectroscopy (^1^H, ^13^C and ^15^N), MS data and elemental analysis. The definite assignment the chemical shifts of protons, carbons and nitrogens (products **4** and **5**) are shown in Table 1, Table 2 and Table 3.

**Table 1:** ^1^H (300 MHz) and ^13^C (75.47 MHz) NMR spectral data for compound **4 **in DMSO-*d6*, including results obtained by homonuclear 2D shift-correlated and heteronuclear 2D shift-correlated HMQC (^1^*J*_CH_)^a^. Chemical shifts (δ, ppm) and coupling constants (*J*, Hz, in parenthesis)^b^.

Position	δ_H_	δ_C_	Correlation ^1^H-^1^H	Correlation ^1^H-^13^C
(NH) **1**	6.29 (s)	-	1-H	-
**2**	-	56.66	-	-
**3**	3.35, 3.45 (AB (2dd), 10.8; 5.6)	64.85	H^1^-3, H^2^-3, O-H	H^1^-3, H^2^-3, C-3
(OH) **4**	4.92 (t, 5.6)	-	O-H, H^1^-3, H^2^-3	-
**5**	1.18 (s)	20.43	H^1^-5, H^2^-5, H^3^-5	H^1,2,3^-5; C-5
**6**	4.35, 4.51 (AB, 14)	51.74	H^1^-6, H^2^-6	H^1^-6, H^2^-6, C-6
**9**	7.95 (s)	151.61	H^3'triazole^ -9	H^3'triazole^ -9, C-9
**11**	8.23 (s)	145.24	H^5'-triazole^ -11	H5'-triazole -11, C-11
**12**	-	154.97	-	-
**14**	-	78.43	-	-
			H^1,2,3^-15	H^1,2,3^-15; C-15
**15; 17; 18**	1.39 (s)	28.68	H^1,2,3^-17	H^1,2,3^-17; C-17
			H^1,2,3^-18	H^1,2,3^-18; C-18

a) Correlation from C to the indicated hydrogens; b) Chemical shifts and coupling constants (*J*) obtained from the 1D ^1^H-NMR spectrum.

**Table 2:** Listing of ^15^N (400 MHz) NMR spectral data for **4 **in DMSO-*d6*, including results obtained by heteronuclear single quantum coherence shift-correlated (**HSQC**) and heteronuclear multiple bond coherence shift-correlated (**HMBC**).

Position	δ_H_	δ_N_	Correlation ^1^H-^15^N
**1** (HN)	7.02 (s)	94.23	H-1,N-1
**5**	1.24 (s)	93.29	H^1,2,3^-5, N-1
**6**	4.39, 4.83 (AB, 14)	93.03	H^1,2^-6, N-1
		214.36	H^1,2^-6, N-7
		299.27	H^1,2^-6, N-8
**9**	7.96 (s)	214.58	H^3'triazole^-9, N-7
		252.01	H^3'triazole^-9, N-10
		299.06	H^3'triazole^-9, N-8
**11**	8.20 (s)	214.58	H^5'-triazole^-11, N-7
		252.01	H^5'-triazole^-11, N-10

Chemical shifts (δ, ppm) and coupling constants (*J*, Hz, in parenthesis) obtained from the 1D ^1^H-NMR spectrum

**Table 3:** ^1^H (300 MHz) and ^13^C (75.47 MHz) NMR spectral data for **5 **in DMSO-*d6*, including results obtained by homonuclear 2D shift-correlated and heteronuclear 2D shift-correlated HMQC (^1^*J*_CH_) ^a^. Chemical shifts (δ, ppm) and coupling constants (*J*, Hz, in parenthesis) ^b^.

Position	δ_H_	δ_C_	Correlation ^1^H-^1^H	Correlation ^1^H-^13^C
(NH)**1**	7.02 (s)	-	1-H	-
**2**	-	58.24	-	-
**3**	-	174.53	-	-
(OH)**4**	12.73 (br, s)	-	-	-
**5**	1.24 (s)	22.33	H^1^-5, H^2^-5, H^3^-5	H^1,2,3^-5; C-5
**6**	4.39, 4.83 (AB, 14)	52.06	H^1^-6, H^2^-6	H^1^-6, H^2^-6, C-6
**9**	7.96 (s)	151.63	H^3'triazole^ -9	H^3'triazole^ -9, C-9
**11**	8.20 (s)	145.56	H^5'-triazole^ -11	H^5'-triazole^ -11, C-11
**12**	-	155.02	-	-
**14**	-	78.95	-	-
			H^1,2,3^-15	H^1,2,3^-15; C-15
**15; 17; 18**	1.40 (s)	28.64	H^1,2,3^-17	H^1,2,3^-17; C-17
			H^1,2,3^-18	H^1,2,3^-18; C-18

a) Correlation from C to the indicated hydrogens; b) Chemical shifts and coupling constants (*J*) obtained from the 1D ^1^H-NMR spectrum.

**Figure 1:**
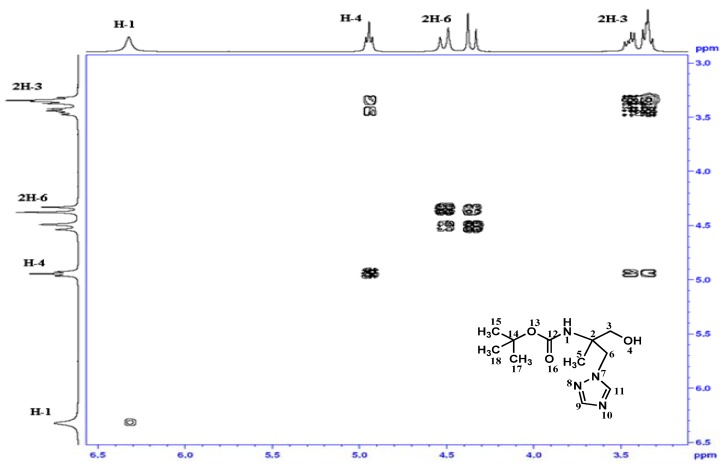
Homonuclear ^1^H-^1^H 2D spectrum for compound **4 **between 3 and 6.5 ppm.

**Figure 2:**
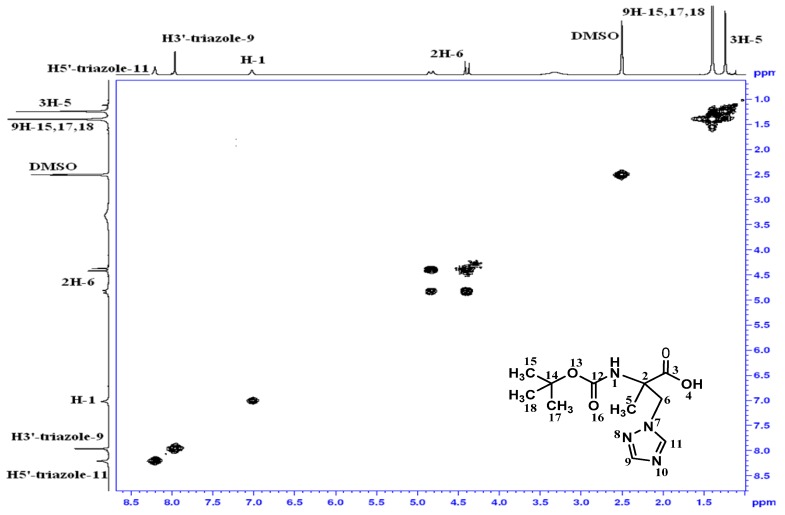
Homonuclear ^1^H-^1^H 2D spectrum for compound **5**.

In the homonuclear ^1^H-^1^H 2D spectra of **4 **(Figure 1) two bond connectivity (^1^*J*_H-H_) between H^1^-6;H^2^-6 and H^1^-3;H^2^-3 can be observed, whereas in the homonuclear ^1^H-^1^H 2D spectra of **5 **(Figure 2), we just observed two bond connectivity between H^1^-6;H^2^-6 and that of H^1^-3;H^2^-3 is absent, indicating the formation of the carboxylic acid. 

**Figure 3:**
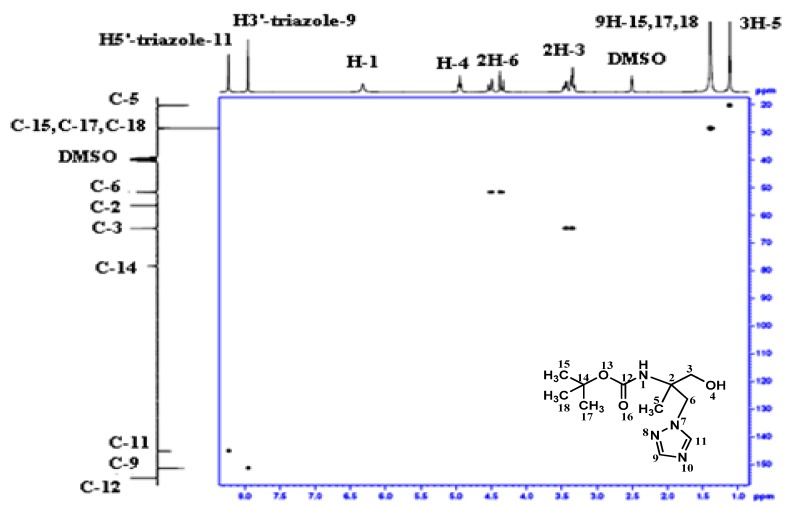
Heteronuclear ^1^H-^13^C 2D spectrum for compound **4**.

**Figure 4:**
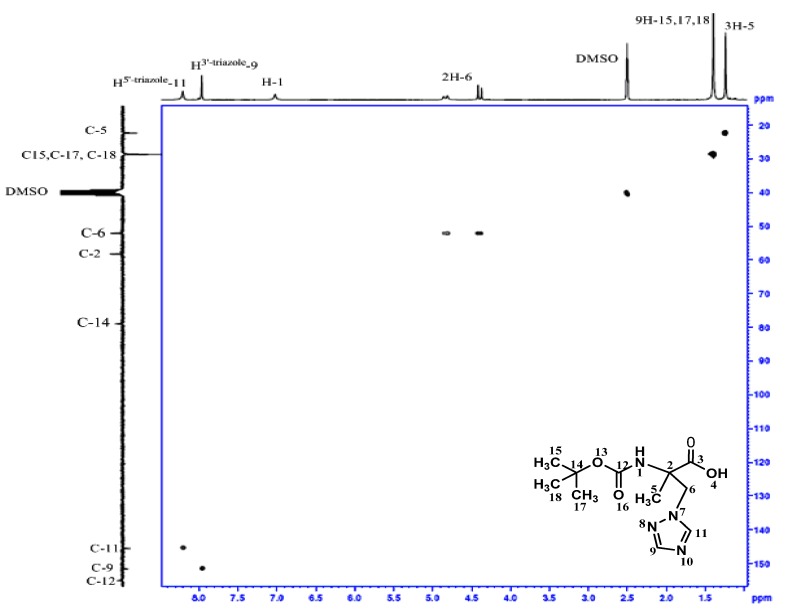
Heteronuclear ^1^H-^13^C 2D spectrum for compound **5**.

**Figure 5:**
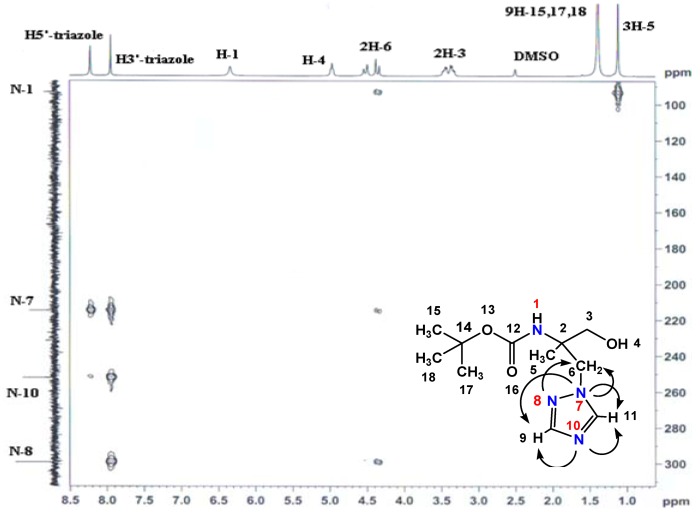
HMBC ^1^H–^15^N spectrum for compound **4**.

In the same way, in the heteronuclear ^1^H-^13^C 2D spectra of **4** (Figure 3), the correlation of C-3 and H^1^-3; H^2^-3 is present, whereas this one is absent in the heteronuclear ^1^H-^13^C 2D spectra of **5 **(Figure 4). Moreover, the carboxyl group resonated at 12.73 ppm and 174.53 ppm in the ^1^H- and ^13^C-NMR spectra of compound **5**. In addition, the analysis of ^15^N NMR spectrum of **4** confirms the N_1_-isomer structure (Figure 5). 

## 3. Experimental

### 3.1. General

All solvents were purified following the standard techniques and commercial reagents were purchased from Sigma Aldrich and Fluka. Melting points were determined with an Electrothermal melting point apparatus and are uncorrected. NMR spectra (^1^H, ^13^C and ^15^N) were recorded on a Bruker AM 300 (operating at 300.13 MHz for ^1^H, at 75.47 MHz for ^13^C and at 30.41 MHz for ^15^N) spectrometer (Centre Universitaire Régional d’Interface, Fez). NMR data are listed in ppm and are reported relative to tetra-methylsilane (^1^H, ^13^C); residual solvent peaks being used as internal standard. All reactions were followed by TLC. TLC analyses were carried out on 0.25 mm thick precoated silica gel plates (Merck Fertigplatten Kieselgel 60F_254_) and spots were visualised under UV light or by exposure to vaporised iodine. Mass spectra were recorded on a PolarisQ Ion Trap GC/MS Mass Spectrometer (Centre Universitaire Régional d’Interface, Fez). Elemental analyses were done in Central Service of Analysis at Rabat. The *O*-tosyl oxazoline derivative **(1)** was prepared in two steps from the commercially available 2-amino-2-methylpropane-1,3-diol using El Hajji’s method [18].

### 3.2. 4-((1H-1,2,4-Triazol-1-yl)methyl)-4-methyl-2-phenyl-4,5-dihydrooxazole *(**2**)*

To a solution of 1*H*-1,2,4-triazole (0.35 g, 5 mmol) in *N*,*N’*-dimethylformamide (12 mL), potassium carbonate (K_2_CO_3_, 0.68 g, 5 mmol) was added by a small portions along with a catalytic quantity of tetra-*n*-butylammonium bromide (TBAB). The mixture is left stirring for 30 minutes, then O-tosyl oxazoline derivative **1 ** (0.35 g, 1 mmol) is added. The reaction mixture was heated to 120 °C for 12 hours with stirring. After cooling, the solvent is evaporated under vacuum and the product was extracted with ethyl acetate and then washed with water. The organic layer was dried on sodium sulfate, concentrated. The oil obtained is purified by column chromatography on silica gel using ether/methanol 5% to afford the pure *N*-alkylated product **2**. Yield 95%; Mol.Wt: 242; R_f_ = 0.31 (ether/ methanol: 9/1); ^1^H-NMR (CDCl_3_, δ ppm): 1.38 (s, 3H, CH_3_); 4.05, 4.59 (AB, 2H, *J* = 8.9 Hz, CH_2_O), 4.31 and 4.37 (AB, 2H, *J* = 14.2Hz, CH_2_N), 7.28-7.82 (m, 5H^arom^), 7.88 (s, 1H^triazole^), 8.17(s, 1H^triazole^). ^13^C*-*NMR (CDCl_3_, δ ppm): 24.82 (CH_3_), 56.97 (1C, 4,5-dihydrooxazole), 70.44(1C, CH_2_-triazole), 75.01(1C, CH_2_ (4,5-dihydrooxazole)), 128.26, 128.43, 131.90, 133.44 (6C, phenyl), 144.32 and 151.42 (2C, triazole), 164.76(1C, 4,5-dihydrooxazole), MS *m/z* (%): 242.99 [M+1] (100), 174.05 (18), 160.07 (10).

### 3.3. 2-Amino-2-methyl-3-(1H-1,2,4-triazol-1-yl)propan-1-ol hydrochloride *(**3**)*

To oxazoline derivative **2 **(1.2 g, 5 mmol) HCl solution (6N, 5 mL) was added and the mixture was refluxed for two hours. After cooling to room temperature, benzoic acid crystals are eliminated by extracting with CH_2_C1_2_, or ether (2 × 25 mL). The aqueous solution is evaporated to a small volume, treated with water, then concentrated to dryness, then washed with a small quantity of ethanol and, finally, again concentrated to dryness. This compound was obtained as colorless oil. Yield 97%; Mol. Wt: 192.1; ^1^H-NMR (DMSO-*d6*, δ ppm): 1.17 (s, 3H, CH_3_); 3.44, 3.49 (AB, 2H, *J* = 11.8 Hz, CH_2_O), 4.52, 4.58 (AB, 2H, *J* = 14.6 Hz, CH_2_N), 5.12 (s, 2H, NH_2_), 8.55 (s, 1H^triazole^), 9.34 (s, 1H^triazole^); ^13^C*-*NMR (CDCl_3_, δ ppm): 18.84 (CH_3_), 52.90 (1C, C(CH_2_OH)), 57.28 (1C, CH_2_-triazole), 63.46(1C, CH_2_OH), 147.19 and 149.49 (2C, triazole); MS *m/z* (%): 192.1 [M] (22), 193.1 (2), 160.1 (100).

### 3.4. tert-Butyl[1-hydroxy-2-methyl-3-(1H-1,2,4-triazol-1-yl)]propan-2-ylcarbamate *(**4**)*

To a cooled (0 < T < 5 °C), solution of aminoalcohol chlorhydrate **3** (1.2 g, 6.3 mmol) in dioxane-water mixture (2/1, 3 mL), triethylamine was added to a neutral pH then Boc_2_O (2.1 g, 8.24 mmol) was added at the same temperature. The whole mixture is taken to room temperature and left under magnetic agitation for two hours. Dioxane was removed and the aqueous phase extracted with ether, then the organic solution is dried over sodium sulphate and evaporated under reduced pressure. The crude product is chromatographed on silica gel using ether/hexane as eluant to afford the pure *N*-protected-β-aminoalcohol **4**. This compound was obtained as a white powder. Yield 80%; Mol.Wt: 256; R_f_ = 0.16 (ether/hexane: 3/1); m.p. = 122–124 °C; ^1^H-NMR (DMSO-*d*6, δ ppm): 1.18 (s, 3H, CH_3_), 1.39 (s, 9H, C(CH_3_)_3_), 3.35, 3.45 (AB (2dd), 2H, *J_AB_* = 10.8 Hz, *J_3_* = 5,6 Hz, CH_2_O), 4.35, 4.51 (AB, 2H, *J* = 14 Hz, CH_2_N), 4.92 (t, 1H, *J_3_* = 5,6 Hz, OH,), 6.29 (s, 1H, NH), 7.95 (s, 1H^triazole^), 8.23 (s, 1H^triazole^); ^13^C*-*NMR (DMSO-*d*6, δ ppm): 20.43 (CH_3_), 28.68 (3C, C(CH_3_)_3_), 51.74 (1C, C(CH_2_OH)), 56.66 (1C, CH_2_-triazole); 64.85 (1C, CH_2_OH), 78.43 (1C, C(CH_3_)_3_), 145.24 and 151.45 (2C, triazole), 154.97 (1C, NHC=O); ^15^N-NMR (DMSO-*d*6, δ ppm): 93.29 (NH-1), 214.31 (N^triazole^-7); 252.08 (N^triazole^-10), 299.06 (N^triazole^-8); MS *m/z* (%) = 257 [M+1] (5), 173.9 (8), 118 (85); 83 (100). Calcd. for C_11_H_20_N_4_O_3 _(%): C 51.55, H 7.87, N 21.86; Found (%): C 51.41, H 7.81, N 21.74.

### 3.5. 2-(tert-Butoxycarbonylamino)-2-methyl-3-(1H-1,2,4-triazol-1-yl)propanoic acid *(**5**)*

To a mixture of *β*-aminoalcohol derivative **4** (0.25 g, 1 mmol) and a solution of sodium hydroxide NaOH (0.12 g, 3 mmol) in water (6 mL) was added a solution of potassium permanganate (0.16 g, 1 mmol) in water (8 mL), under vigorous stirring during 4 hours. The mixture was cooled to 4–5 °C by immersion in a bath of ice water, and then the reaction mixture was allowed to gradually attain room temperature. After 12 hours, the precipitate manganese dioxide was filtered off and then the filtrate was cooled. The solution was covered with a layer of ethyl acetate and acidified with dilute sulfuric acid. The ethyl acetate layer was separated and the aqueous layer was extracted three times with ethyl acetate (25 mL). The combined ethyl acetate extracts were dried over anhydrous sodium sulfate. Finally, the ethyl acetate was then removed on a rotavapor. This compound was obtained as a white powder. Yield 92%; Mol.Wt: 270; R_f_ = 0.08 (ether); m.p. = 188–190 °C; ^1^H-NMR (DMSO-*d*6, δ ppm): 1.24 (s, 3H, CH_3_), 1.40 (s, 9H, C(CH_3_)_3_), 4.39, 4.83 (AB, 2H, *J* = 14 Hz, CH_2_N), 7.02 (s, 1H, NH)), 7.96 (s, 1H^triazole^), 8.20 (s, 1H^triazole^), 12.73 (br, 1H, COOH); ^13^C-NMR (DMSO-*d*6, δ ppm): 22.33 (CH_3_), 28.64(3C, C(CH_3_)_3_), 52.06 (1C, C(COOH)), 58.24 (1C, CH_2_-triazole), 78.95 (1C, C(CH_3_)_3_), 145.56 and 151.63 (2C, triazole), 155.02 (1C, NHC=O), 174.53(1C, COOH); MS *m/z* (%): 271 [M+1] (10), 154.1 (24), 83 (100). Calcd. for C_11_H_18_N_4_O_4 _ (%): C 48.88, H 6.71, N, 20.73; Found (%): C 48.76, H 6.57, N 20.71.

## 4. Conclusions

In conclusion, this work describes the synthesis of a novel heterocycle-substituted amino acid based on using an oxazoline as a masked amino acid. The *N*-alkylation of 1,2,4-triazole with *O*-tosyl derivative **1** was occurred under very mild conditions. The regioselectivity was excellent, and only the N_1_-isomer was obtained.
